# COVID-19 Induced Bilateral Lower Limb Ischemia and Visceral Infarcts

**DOI:** 10.7759/cureus.12784

**Published:** 2021-01-19

**Authors:** Spandana Narvaneni, Sydney M Fasulo, Vinod Kumar, Balraj Singh, Yasmeen Sultana

**Affiliations:** 1 Internal Medicine, St. Joseph's Regional Medical Center, Paterson, USA; 2 Hematology and Oncology, St. Joseph's Regional Medical Center, Paterson, USA; 3 Infectious Disease, St. Joseph's Regional Medical Center, Paterson, USA

**Keywords:** covid19, coronavirus, thrombosis, hypercoagulability, limb ischemia, renal infarcts

## Abstract

Emerging cases of coronavirus disease 2019 (COVID-19) caused by the severe acute respiratory syndrome coronavirus 2 (SARS-COV-2) have been associated with a variety of disorders including respiratory failure, immune reactive syndrome, multiorgan failure, and hypercoagulable states. COVID-19 induces a severe global inflammatory response which can result in endothelial damage leading to hypercoagulability. Most COVID-19 cases of hypercoagulable states reported venous thrombosis. We report here a case of a 65-year-old Hispanic male diagnosed with bilateral acute lower limb ischemia and renal infarcts secondary to a severe COVID-19 infection.

## Introduction

There is increasing knowledge of coronavirus disease 2019 (COVID-19) hypercoagulability with several case reports of venous thrombosis, however, there is limited information on arterial thrombosis [1]. In this case, we demonstrate the development of multiple arterial thrombotic events including renal infarcts and acute limb ischemia, as well as venous thrombi with multiple pulmonary emboli.

## Case presentation

A 63-year-old Hispanic male with no past medical history presented to the ED for shortness of breath, dry cough, and worsening fatigue for approximately one-week duration. He was hypoxic on presentation saturating 88% on room air. 

**Figure 1:**
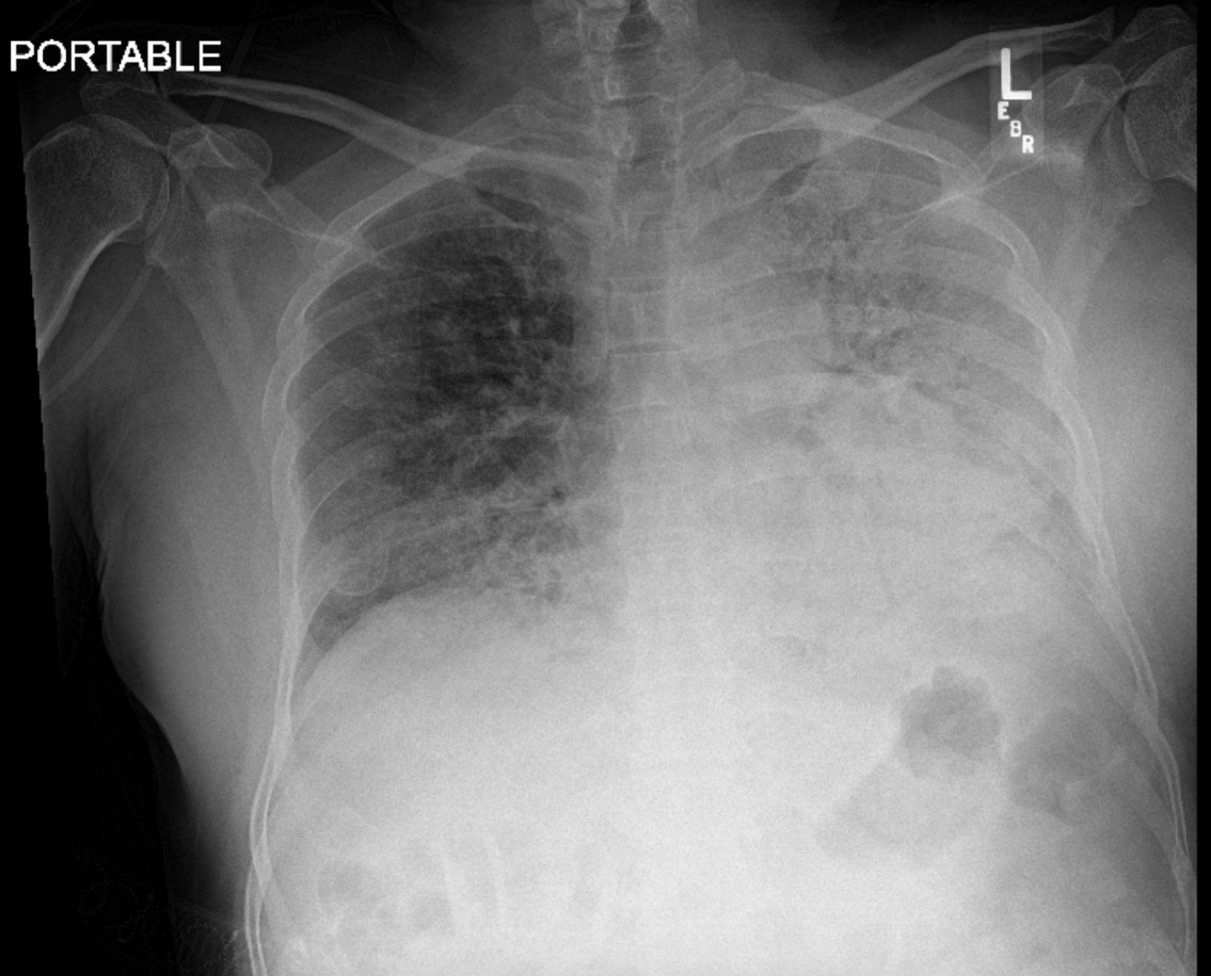
Portable AP chest X-ray demonstrating pan lobar infiltrates with more confluent opacity in the lower lungs, worse on the left than the right. AP, anteroposterior

Chest X-ray demonstrated bilateral hazy infiltrates, greater in the left lung field (Figure 1). Laboratory evaluation showed elevated inflammatory markers including a lactate dehydrogenase of 558 U/L, ferritin of 1218 µg/L, erythrocyte sedimentation rate 57 mm/h, C-reactive protein of 133.8 mg/dL, d-dimer of 0.6 µg/mL, elevated liver enzymes, serum creatinine of 0.9 mg/dL which later normalized to 0.4 mg/dL, and elevated white blood cell count 15,000/mm3. The severe acute respiratory syndrome coronavirus 2 (SARS-COV-2) polymerase chain reaction testing was positive. 

The patient was started on IV dexamethasone, ceftriaxone, azithromycin, and remdesivir. A nasal cannula at 3 L was not providing satisfactory oxygenation and the patient was switched to a nonrebreather at 10 L and then to bilevel positive airway pressure. 

**Figure 2:**
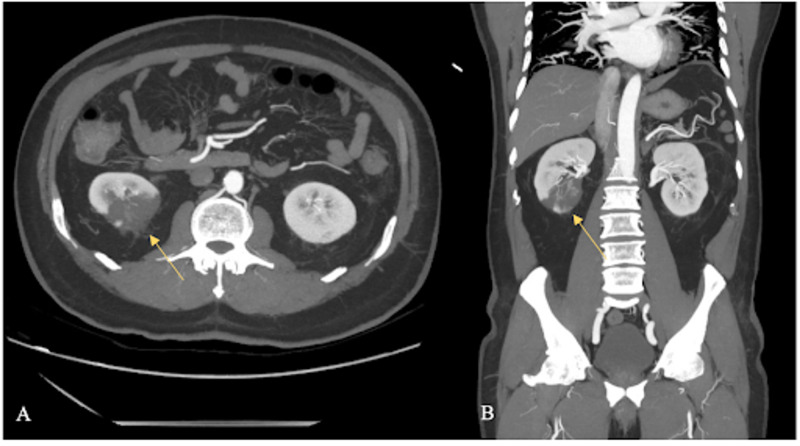
Axial image (A) and coronal image (B) from the arterial phase of a CTA demonstrating wedge-shaped regions of hypoenhancement within the lower pole of the right kidney. CTA, computed tomography angiogram

**Figure 3:**
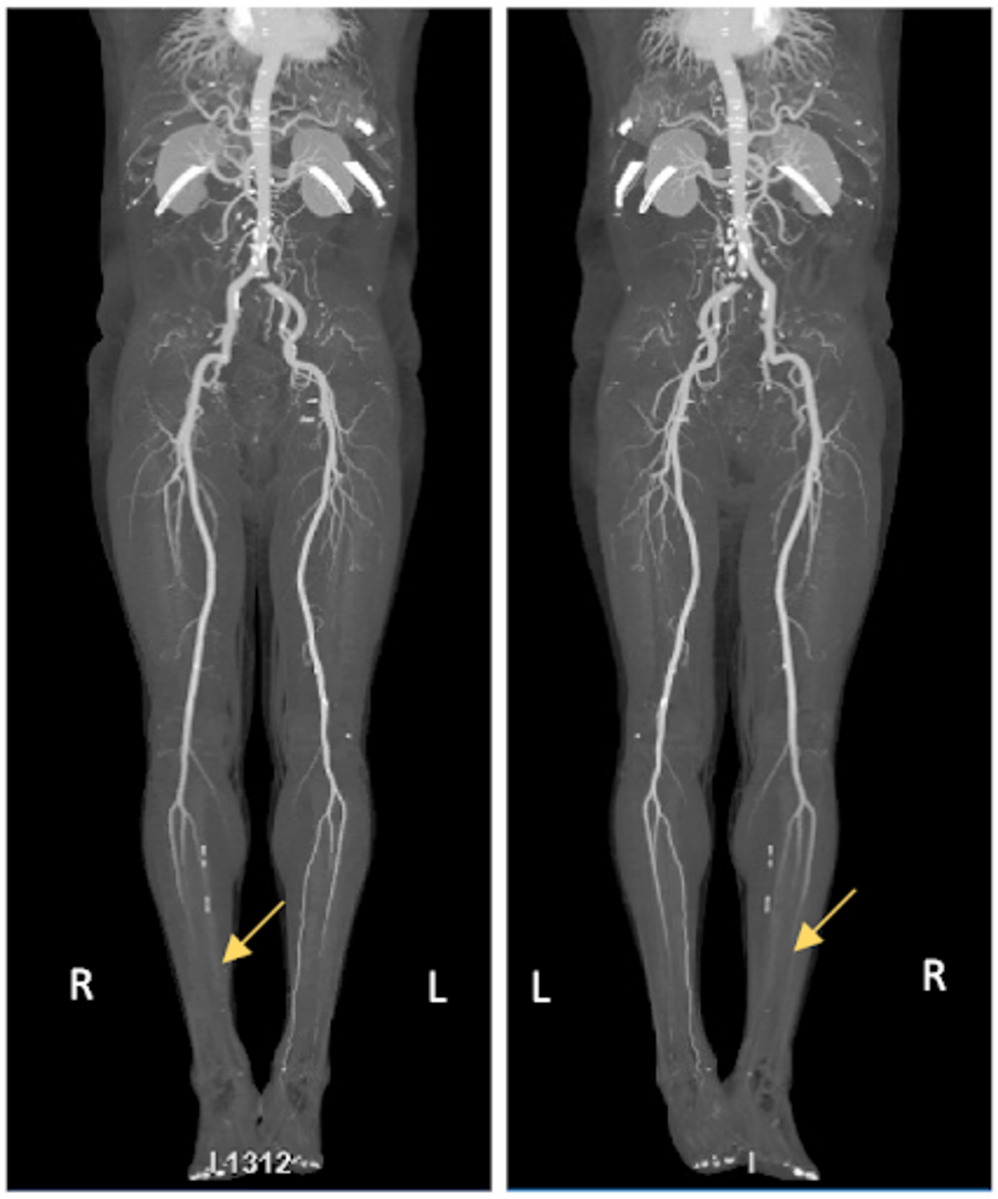
Anterior and posterior images from the CTA with iliofemoral run-off showing decreased patency of the vessels of the lower extremities, right worse than left. CTA, computed tomography angiogram

On day 3 of admission, the patient reported right lower extremity pain and was found to have absent dorsalis pedis and posterior tibial pulses. He was also in increased respiratory distress and tachypneic at which point he was placed on a high-flow nasal cannula. With concern for multiple arterial thrombi, a computed tomography angiogram (CTA) with iliofemoral runoff was ordered. The CTA demonstrated right middle and lower lobe pulmonary emboli, a large wedge-shaped region of hypoenhancement in the right kidney (Figure 2) and smaller wedge-shaped regions of hypoenhancement within the left mid to lower kidney representing renal infarcts. The run-off showed moderate stenosis within the origin of the right peroneal artery and segmental occlusion of the left peroneal artery (Figure 3).

 

 

The patient was given an IV heparin bolus and started on an IV heparin drip. The patient and family decided not to proceed with vascular surgical intervention. A 2D echo was obtained to look for a cardiac source of emboli and resulted normally. Transesophageal echocardiogram with a bubble study, though useful to rule out patent foramen ovale, was unable to be performed as the patient later became unstable and required intubation and mechanical ventilation for continued hypoxia on noninvasive modes of ventilation. No cardiac arrhythmias were observed during his hospital course.

A hypercoagulable workup was negative including anti-phospholipid antibodies; beta 2-microglobulin, anticardiolipin, and lupus anticoagulant. The patient was maintained on therapeutic anticoagulation with enoxaparin sodium. 

## Discussion

With the continued rise of COVID-19 infections, we continue to learn more about COVID-19 infection. Since the start of the COVID-19 pandemic, we have learned that infected patients are at increased risk for thrombotic events. A recent review of the literature showed patients with COVID-19 infections are at increased risk for deep venous thrombosis, pulmonary embolism, cerebral infarction, and visceral infarction [1-2]. 

Very little is known about the mechanism of the hypercoagulable state which develops secondary to COVID-19 infections. Two proposed mechanisms are disseminated intravascular coagulation (DIC) and endotheliopathy [3]. COVID-19 infection induces a global systemic inflammatory response which releases cytokines and stimulates the coagulation cascade. The product of this cascade activation causes the formation of fibrin clots resulting in DIC. Endotheliopathy occurs when the SARS-CoV-2 virus binds to the endothelial cells of the blood vessels using the angiotensin-converting enzyme 2 receptor. The virus then replicates, causing an infiltration of inflammatory cells that induces endothelial cell apoptosis, which leads to a hypercoagulable state.

Although we continue to learn about the significant associated thrombotic events in COVID-19 patients there are still only a few case reports of COVID-19 induced renal infarction, such as Ammous et al. [3] who reported one case and Post et al. [4] who reported two cases. There are even fewer reports of COVID-19 associated limb ischemia [5-8]. 

## Conclusions

We are reporting here a case of a 65-year-old Hispanic male with COVID-19 associated renal infarction and bilateral acute lower limb ischemia which has been only rarely reported, particularly in a middle-aged patient with no comorbidities. Awareness of the presence of arterial thrombi is an important consideration in the management and treatment of severe COVID-19 infections.
